# Book Reviews

**Published:** 1885-06

**Authors:** 


### BOOK REVIEWS.

A TEXT-BOOK OF PATHOLOGICAL ANATOMY AND PATHOGENESIS. *By* ERNST ZEIGLER, *Professor of Pathological Anatomy in the University of Tübingen. Translated and Edited for English Students by* DONALD MACALISTER, M. A., M. B., *Member of the Royal College of Physicians; Fellow and Medical Lecturer of St. John's College, Cambridge. Part II.—Special Pathological Anatomy. Sections I.—VIII. London:* MACMILLAN & CO. 1884.

This, the second part of Zeigler's Pathology, speaks of special pathological anatomy, divided into eight sections. Section I. is devoted to the consideration of the Blood and Lymph, the various pathological conditions to which they may be subjected is referred to in a general manner in the first chapter. The following chapters then treat in a clear and concise manner of the special pathological alterations, as changes in the chemical composition, changes in the quantity and composition of the blood. Under this head are classified plethora, amæmia, oligocythæmia and other conditions dependent upon the quantitative and qualitative changes in the blood. The other sections which refer to the vascular system, spleen and lymphatic glands, serrus membranes, skin, mucous membranes, alimentary tract, liver and pancreas, are all treated in the same systematic and classic manner.

Special mention should be made of the excellent, truly artistic manner in which the wood-cuts have been executed. They surpass anything of a similar nature found in text-books.

They assist very materially in increasing the value of the work. The book reads as if originally written in the English language. Its general appearance is very good, the paper is of good quality, and the print, with the exception of the notes, is large and distinct. The headings of chapters and paragraphs are printed in large capitals, which enables the reader to find, at a glance, any subject of special interest. B. B.

---

PRACTICAL PATHOLOGY. A MANUAL FOR STUDENTS AND PRACTITIONERS. *By* G. SIMS WOODHEAD, M. D., F. R. C. P. E., *Demonstrator of Pathology in the University of Edinburgh ; Pathologist to the Royal Hospital for Sick Children; late President of the Royal Medical Society, etc., etc. With One Hundred and Thirty-six Colored Plates. Philadelphia:* HENRY C. LEA'S SON & CO. *1884.*

The author of this work has fully realized the deficient manner in which pathology is taught in our schools, the scant opportunity offered students to acquaint themselves with the macroscopic and especially microscopic appearance of healthy and diseased tissues. A thorough understanding of pathological processes can only be obtained by the formulation in the mind of a distinct picture of the changed appearance of the tissues. He who has never viewed through a microscope, or seen a graphic description of a cirrhotic liver, can as little obtain a clear and intelligent conception of such a process as he could of the construction of a chronometer from a verbal description. The author has attempted to substitute for the lack of practical instruction a number of illustrations depicting the most typical changes found in various pathological conditions.

The book also serves as a guide to laboratory work. The first few chapters, devoted to post mortem examinations and microscopic technology, are tolerably complete and practical. All known staining methods, including the quite recent formulæ for bacteria staining, are mentioned.

The remainder of the book deals with the most common pathological changes of the various tissues of the body. The work is modeled after the plan of Frey's.

We can recommend the work for its clearness of style, brevity and completeness. B. B.

---

AN INTRODUCTION TO PATHOLOGY AND MORBID ANATOMY. *By* J. HENRY GREEN, M. D., *Fellow of the Royal College of Physicians, London, etc., etc. The fifth American, from the sixth revised and enlarged English edition, with one hundred and fifty engravings. Philadelphia:* HENRY C. LEA'S SON & CO. 1884.

Henry Green's text book on Pathology is well known to the English-speaking medical public. It has always been the object of the author to represent the present state of our knowledge. To meet this demand, several chapters have been added by Mr. Stanley Boyd, which improve the value of the book. The chapter on " Karyokimsis," forms a fitting introduction to the volume, founded on the dictum of Virchow—*Omnis cellula e cellula.* The chapter on vegetable parasites contains a general *résumé* of the Germ Theory, started by Astur, Schwann and Cagnaird de Latour, and advanced by Pasteur's researches. The physical theory originated by Willis and upheld by Liebig, is discussed in its various bearings. The author concludes that all the processes generally known as fermentation and putrefaction are due to the action of vegetable organisms. The natural history of the vegetable parasites, also the researches of Koch, Klebs, Pasteur, Cohen, Nieser and others, are briefly mentioned, thus giving the student, in brief, the results of years of scientific labor, accounts of which are scattered throughout our literature. The fact that the work has passed through a fifth edition, may be taken as evidence of its excellency and of its appreciation by the medical world. B. B.

---

A MANUAL OF ORGANIC MATERIA MEDICA. *By* JOHN M. MAISCH. *Philadelphia:* LEA BROS. & CO. *Chicago:* JANSEN, McCLURG & CO.

That portion of Materia Medica relating to inorganic substances need necessarily differ so little from the subject of inorganic chemistry, that the student is usually able to grasp it without much difficulty. Here a scientific classification is possible, and the substances having, in general, a fixed and known composition, both teacher and student feel that they are walking on well-defined ground. As much cannot be said of the organic bodies used in medicine. In most cases it is not even possible to refer them to any one of the more or less complex groups now recognized in organic chemistry, as their composition is often quite variable, not always admitting of easy determination. This fact increases very greatly the difficulty which the student must encounter in a study of the subject. A strictly scientific classification being out of the question, writers follow various arbitrary plans. Some have arranged substances in an alphabetical order; others arrange them according to the purposes for which they are used in medicine; but this method, while it possesses advantages for the student, must be characterized as very unscientific, and, at best, only provisional, as it throws bodies together which have merely this in common: that as remedies, they have certain resemblances. The therapeutics of another generation might suggest an entirely different classification.

The work before us, which appears now in its second edition, and is intended for pharmacists rather than for physicians, follows another system. Organic drugs are divided into three groups, viz., those of animal origin, those of vegetable origin having cellular structure, and those of vegetable origin without cellular structure. Of the animal drugs, six sub-groups are made; of the cellular vegetable, twelve, depending chiefly on botanical features, and of the vegetable drugs without cell structures, six, depending chiefly on chemical properties.

Under each heading the important physical and chemical characteristics are given, and usually fully enough for identification. Many of the articles are furnished with illustrations, adding greatly to the practical value of the work. Its size, about 500 pages, is such that it can be used as a class book in connection with a course of lectures, and for this purpose it is undoubtedly the best manual we have. J. H. L.

---

DISEASES OF THE NOSE. *By* CLINTON WAGNER, M. D., *Professor of the Diseases of the Nose and Throat in the New York Post-Graduate School, etc., etc. With Illustrations of Instruments and Pathological Conditions. Octavo, pp. 252, New York;* BIRMINGHAM & COMPANY.

Rhinoscopy has now attained the age of a quarter of a century. Its discovery opened up fresh territory in the domain of medicine, some of the fruits of which are exemplified in the many manuals and treatises upon diseases of the nose, which have appeared during the past decade, while the ready sale enjoyed by most of these books, is evidence as well of their merit as of the wide-spread interest in the subject. The present work by Professor Wagner, is deduced from an extensive experience and well deserves our favorable consideration.

The preliminary chapter upon the anatomy of the Nasal Fossae is illustrated by fair wood-cuts, which save much time and endless annoyance in reference, and the authors of some treatises of greater pretentions might well "take the hint" in the preparation of their future editions.

On page 21, occurs the statement: "This so-called superior turbinated bone protrudes so slightly into the nasal fossa, that it cannot be seen on the living subject, either through the anterior or posterior nares," language certainly inconsistent with a cut upon page 49, of the rhinoscopic image of the posterior nares, in which the superior turbinated bone is figured. This body is frequently perceptible in the rhinoscopic mirror, although it is not always visible.

In view of the frequent assertions, by those unaccomplished in the art, of the impracticability of posterior rhinoscopic examination, it is interesting to learn that the author succeeded, thoroughly, in so examining ninety-six *per cent.* of a continuous run of hospital cases.

Fresh-air maniacs, those who insist upon sleeping during the inclement months, with widely opened windows, should note, that this practice is regarded as a "not unfrequent" exciting cause of chronic nasal catarrh, and we would further add that to our certain knowledge it is a most prolific source, also, of *acute* laryngeal, pharyngeal and nasal inflammations. It is certainly unreasonable to pass the day in, perhaps, superheated apartments and the night, when animal vitality is at lowest ebb, in an atmosphere cold and damp, and in our climate, often of a temperature below zero.

In the treatment of nasal stenoses from deflection of the septum, enchondroma, or osteoma, Professor Wagner advocates the removal of the obstructing parts by rapidly revolving knives and burrs driven by the dental engine. No anæsthetic is requisite as the very rapid revolutions of the burr cause comparatively little pain. The results in the cases which he reports are exceedingly satisfactory,

The danger to life from the removal of naso-pharyngeal fibroma, by the galvano-cautery ecraseur, is stated to be "absolutely none," which recalls to our mind a case witnessed, a few years since, at the *Allgemeines Krankenhaus* in Vienna. A robust young man with a naso-pharnygeal fibroma the size of a walnut; removal *ad secundum artem* with the galvano-cautery écraseur, by Professor Schötter; death within one week from pyæmia; autopsy: suppurative pericarditis and metastatic abscesses. Such a deplorable termination is, however, the rarest of occurrences. The phraseology of the book is clear and concise, the paper and print are very good, and typographical errors are few in number.

W. E. Casselberry.

---

A Hand-Book of Pathological Anatomy and Histology. *By* Francis Delafield, m. d., and T. Mitchell Prodden, m. d. Wm. Wood & Co., *New York*. *pp. xvi.—575.*

This is the second volume of Dr. Delafield's "Hand-Book of Post Mortem Examinations and Morbid Anatomy," and is issued in conjunction with Dr. Prodden.

The work comprises four parts :—

1st. The method of making post mortem examinations, and of preserving diseased tissues and preparing them for microscopical study.

2d. Morbid changes in the circulation of the blood.—Changes in the composition of the blood.—Degenerative changes in the tissues.—Animal parasites.—Bacteria, their relation to disease and the method of preparing and examining them.—Inflammatory and allied changes in tissues.—A classification and description of tumors.

3d. The macroscopic and microscopic morbid anatomy of organs.

4th. Lesions found in the general diseases, in poisoning and in violent deaths.

The subject matter is systematically arranged and clearly treated, and aside from valuable additions, the book has been improved by revision.

The illustrations greatly enhance the usefulness of the work, and are of the first order of excellence.

The book will be found valuable as a Hand-Book of Diagnosis in Pathological Anatomy.

F. S. J.

---

Intestinal Obstruction: Its Varieties, with their Pathology, Diagnosis and Treatment. *The Jacksonian Prize Essay of the Royal College of Surgeons of England. 1883. By* Frederick Treves, F. R. C. S. *60 Illustrations.* Henry C. Lea's Son & Co., *Philadelphia;* Jansen, McClurg & Co., *Chicago. 120 pp. x.—515.*

The limited space usually assigned to the subject of intestinal obstruction in our systematic works of reference, makes the appearance of this little book especially acceptable to all who appreciate thorough investigation and are willing to forego, in a large measure, theoretical considerations.

The different varieties of intestinal obstruction are classified upon a pathological basis, and are considered under the following heads:

Strangulation by bands, etc., or through apertures.

Volvulus.

Intussusception.

Stricture.

Obstruction by neoplasms.

Compression by tumor, etc., external to the bowel.

Obstruction by gall-stones and foreign bodies.

" " enteroliths.

" " fæcal masses.

The pathology, symptomatology, cause, and prognosis of each is treated in turn.

In the latter chapters on diagnosis the subject is considered from the clinical standpoint under the headings:—

1. The general significance of the leading symptoms.

2. The diagnosis of the different forms of intestinal obstruction.

3. The symptoms as modified by the position of the obstruction.

4. The various affections that have been most frequently confused with cases of obstruction of the bowels.

Lastly: three chapters on treatment, discussing non-operative and operative measures.

Throughout the work, there is constant reference made to illustrative cases either observed by the author or recorded by others; and to the pathological specimens preserved in museums.

Interesting facts and cases, published since the completion of the essay, have been incorporated in the work.

The book appears in the convenient form of the clinical manuals issued by the publishers. F. S. J.

STUDENT'S MANUAL OF HISTOLOGY. *For use of Students, Practitioners and Microscopists. 3rd Edition. Entirely re-written, greatly enlarged and newly illustrated. 78 Engravings. By* CHARLES H. STOWELL, M D., F. R. M. S. *University of Michigan, Ann Arbor, Michigan. Published by the Author. 8vo. pp. 368.*

The introductory chapters—I. and II.—briefly describe the microscope, its principal accessories, and their uses, and such re-agents and methods of preparing animal tissues as the author has found most useful.

The chapters on the microscopic anatomy consist of short descriptions of the tissues and organs of the body. An effort is also made to lay before the reader briefly the results of recent investigations. Closing each chapter is given a section indicating the special methods best adapted to the study of the tissue described.

The last two chapters are devoted respectively to short descriptions of tumors and of the various starches met with as adulterants of commercial articles.

The work will perhaps be found more useful as a manual for the laboratory than as a text-book for the class-room.

We hope that if another edition should be issued, the author will take the opportunity to adopt a more logical and scientific arrangement of subjects, and will omit extraneous matter; in a word, that he will make the book what it should be,—a concise manual of normal histology. F. S. J.

